# Science and application of strigolactones

**DOI:** 10.1111/nph.16489

**Published:** 2020-03-17

**Authors:** Ernest B. Aliche, Claudio Screpanti, Alain De Mesmaeker, Teun Munnik, Harro J. Bouwmeester

**Affiliations:** ^1^ Plant Hormone Biology Swammerdam Institute for Life Sciences University of Amsterdam Science Park 904 Amsterdam 1098 XH the Netherlands; ^2^ Chemical Research Syngenta Crop Protection AG Schaffhausenstrasse 101 CH‐4332 Stein Switzerland; ^3^ Plant Cell Biology Swammerdam Institute for Life Sciences University of Amsterdam Science Park 904 Amsterdam 1098 XH the Netherlands

**Keywords:** agriculture, application, microbiome, stress, strigolactones

## Abstract

Strigolactones (SLs) represent a class of plant hormones that regulate developmental processes and play a role in the response of plants to various biotic and abiotic stresses. Both *in planta* hormonal roles and *ex planta* signalling effects of SLs are potentially interesting agricultural targets. In this review, we explore various aspects of SL function and highlight distinct areas of agriculture that may benefit from the use of synthetic SL analogues, and we identify possible bottlenecks. Our objective is to identify where the contributions of science and stakeholders are still needed to achieve harnessing the benefits of SLs for a sustainable agriculture of the near future.

## Introduction

**Fig. 1:**
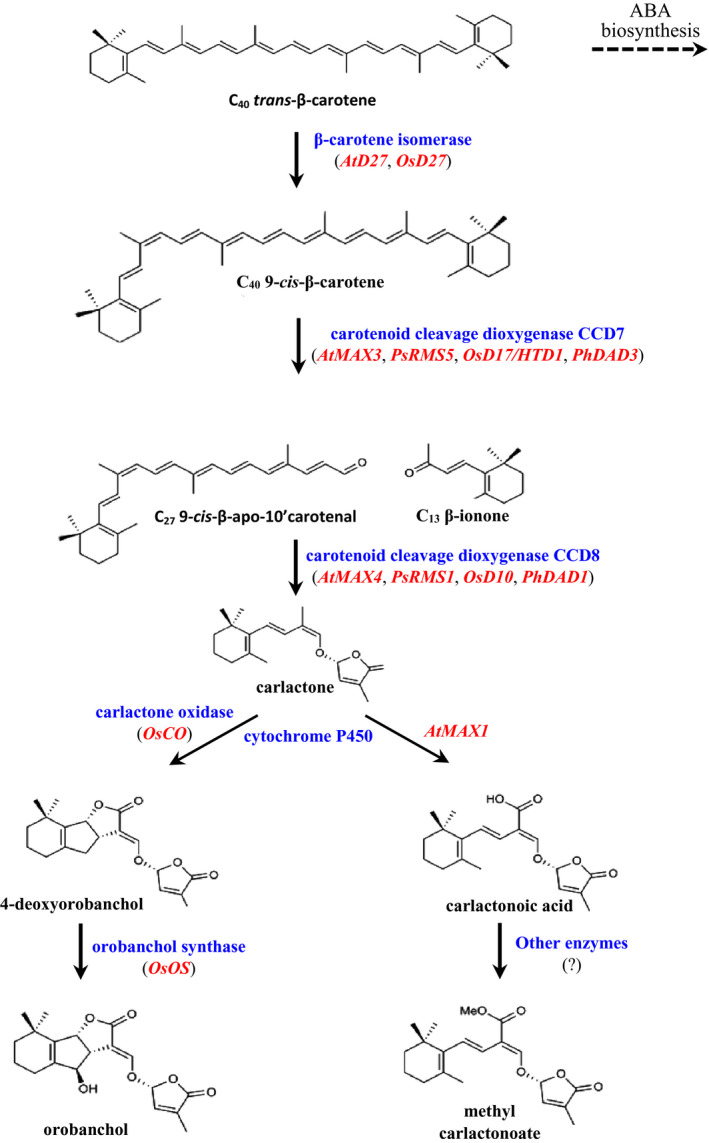
Strigolactone biosynthesis pathway showing the substrates (in black), proteins (in blue), and genes (in red) encoding the enzymes involved in Arabidopsis (*At*), rice (*Os*), pea (*Ps*), and petunia (*Ph*) (Flematti *et al.*, 2016). Abscisic acid (ABA) biosynthesis competes with strigolactone biosynthesis for *all*‐*trans*‐β‐carotene as substrate.

**Fig. 2:**
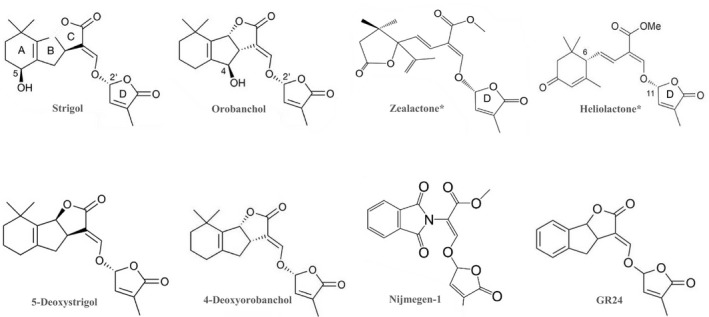
Structural variation in natural and synthetic strigolactones (SLs). All these SLs have the conserved D‐ring (see strigol). The canonical SLs have the full ABC‐ring system, as in strigol, orobanchol, 5‐deoxystrigol, and 4‐deoxyorobanchol. The noncanonical SLs have an incomplete ABC‐ring system, as illustrated with zealactone and heliolactone (marked with asterisk). 5‐deoxystrigol and 4‐deoxyorobanchol illustrate the differences in orientation of the BC ring junction. Nijmegen‐1 and GR24 are examples of synthetic SLs. Adapted from Wang & Bouwmeester (2018) and Yoshimura *et al.* (2019).

Strigol, the first strigolactone (SL), was isolated in 1966 from cotton root exudate (Cook *et al.*, 1966), yet it took more than 40 years to realize that SLs represent a new class of phytohormones (Gomez‐Roldan *et al.*, 2008; Umehara *et al.*, 2008). Despite the time lapse between discovery, elucidation of its structure, and recognition as a hormone, the recent rise in research focus on SLs suggests a promising future for this class of signalling molecules (Cook *et al.*, 1972; Zwanenburg & Blanco‐Ania, 2018). This review introduces the prospects of such a future by highlighting the science of SLs and their potential application in agriculture. SLs are derived from β‐carotene (Alder *et al.*, 2012). Partial elucidation of their biosynthesis in several plant species has identified the involvement of the following genes: *DWARF27* (β‐carotene isomerase), *CAROTENOID CLEAVAGE DIOXYGENASE 7* and *8* (CCD7 and CCD8), and *MAX1* homologues (cytochrome P450s) (Lopez‐Obando *et al.*, 2015; Fig. 1). According to recent reviews, more than 25 SLs have been identified across the plant kingdom, categorized into canonical and noncanonical SLs based on the presence or absence, respectively, of the complete ABC‐ring system (Wang & Bouwmeester, 2018; Bürger & Chory, 2020). A conserved feature in both canonical and noncanonical SLs is the D‐ring, but otherwise many structural variations – including differences in stereochemistry and lack of the conventional ABC‐ring system (noncanonical SLs) – have been reported (Wang & Bouwmeester, 2018; Fig. 2). A butenolide structure similar to the SL D‐ring is also found in smoke‐derived signalling molecules, karrikins, which have been implicated in the germination of dormant seeds after a bush‐fire (Flematti *et al.*, 2015). Karrikins are presumed to mimic an as yet‐unknown endogenous signalling molecule involved in early plant development, and share a paralogous signalling pathway with the SLs (Flematti *et al.*, 2015).

Synthetic SLs are an important tool in the biological research on the functions of these signalling molecules. Chemical synthesis involves either a total synthesis of the entire SL structure or synthesis of analogues with a simplified structure that retains the bio‐properties of SLs (Zwanenburg *et al.*, 2015; Oancea *et al.*, 2017). Total synthesis of the ABC scaffold and subsequent attachment of the functional side‐chains and D‐ring is tedious, and yield is low (Zwanenburg *et al.*, 2015). Chemical synthesis of SL analogues is more promising and is feasible based on the identification of the bioactiphore in SLs, the D‐ring, which is required for activity. Although the contribution of the A‐ring to activity is low, it should have the required stereochemistry (molecular freedom) to get reasonably active analogues (Zwanenburg *et al.*, 2015). A report has shown that structural modification of the D‐ring into a γ‐lactam functional group may give insight into the variations in SL binding interaction with its receptor (Lombardi *et al.*, 2017). Another important analogue is the fluorescence turn‐on probe, Yoshimulactone Green, which can be used to track SL perception (Tsuchiya *et al.*, 2015). All these synthetic SLs have greatly contributed to improve our understanding of the biological role of SLs. However, SL synthesis is faced with a number of challenges (see Box 1).

Box 1 **Synthetic production and regulatory bottlenecks to strigolactone application**

Having highlighted the potential that lies in the application of strigolactones (SLs) in agriculture, an inevitable prerequisite for harnessing such potential is that synthetic SL products need to be developed. Some of the synthetic SLs that have been produced so far include GR24 (Besserer *et al*., 2008), Nijmegen‐1 (Nefkens *et al*., 1997), Strigolactams (De Mesmaeker *et al*., 2019), and the fluorescent EGO‐15 and ST‐23b (Prandi *et al*., 2011), sphynolactone‐7 for *Striga* control (Uraguchi *et al*., 2018), and CISA‐1 (Rasmussen *et al*., 2013) (Fig. 2). The core bioactivity of these synthetic SLs mainly depends on the presence of the D‐ring, although the side‐chain functional groups do modify SL function and activity (Boyer *et al*., 2012).

One of the challenging requirements for synthetic SLs is their stability in solvent media in which they are formulated, as this contributes to efficacy, which may be negatively affected by environmental conditions, such as temperature and pH (Zwanenburg & Pospíšil, 2013). Furthermore, these SL products must meet the relevant regulations that are in place to ensure their environmental friendliness and safety. For instance, transportability to target tissues and rapid hydrolysis after its action has been initiated.

In terms of regulatory requirements for agricultural end‐uses, SL products may be categorized either as plant protection products, plant strengtheners, or plant bio‐stimulants during product registration procedures, and such diversified categorization may make it hard for regulatory bodies to have a definitive regulation in the use of SL products (Vurro *et al*., 2016). Additionally, overall investment to optimize and develop a new synthetic agrochemical costs *c*. USD 300 million (Syngenta Crop Protection AG, 2016), and the years involved in the application procedure without a guarantee of return on investment may dissuade industrial partners from commercializing synthetic SLs (Vurro *et al*., 2016). Despite the aforementioned requirements and complexities, a number of industries have already been investing significant resources into SL‐based agricultural trials (Davidson, 2015; Screpanti *et al*., 2016). These efforts may contribute to unveiling the usefulness of SLs to all stakeholders and possibly ease their registration, for instance, as plant strengtheners or plant bio‐stimulants.

**Fig. 3:**
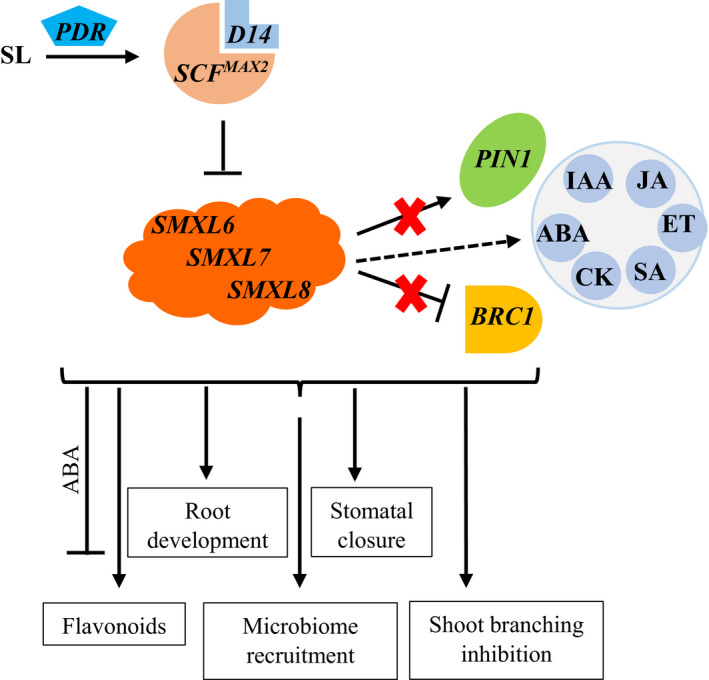
Model of strigolactone (SL) signalling and downstream physiological and phenotypic effects. The dashed arrow indicates the complex inter‐hormonal interactions of SLs. ABA, abscisic acid; CK, cytokinin; ET, ethylene; IAA, auxin; JA, jasmonic acid; SA, salicylic acid.

The structural variations in the SLs are reflected in their functional diversity (Scaffidi *et al.*, 2014). For instance, differences in the effects of various SLs and their stereoisomers on the germination of parasitic weeds, such as *Striga hermonthica* and *Striga gesnerioides*, could be attributed to their structural variation (Nomura *et al.*, 2013). Using germination assays, Nomura *et al.* (2013) showed that SLs that have the same configuration as 5‐deoxystrigol at their C3a, C8b and C2 positions triggered high germination of *S. hermonthica* but not *S. gesnerioides*. Furthermore, the recognition of natural SLs and nonnatural SL isomers (and karrikins), which is mediated by the receptors D14 and KAI2, respectively, is dependent on the structural variations in the chiral carbon orientations at the junction of the BC and D‐rings (Scaffidi *et al.*, 2014). This specificity in SL recognition was demonstrated in Arabidopsis using 5‐deoxystrigol, which showed active, D14‐dependent, inhibition of hypocotyl elongation but not KAI2‐dependent seed germination (Scaffidi *et al.*, 2014). Both of the specific receptor molecules (D14 and KAI2) use MORE AXILLARY GROWTH2 (MAX2) for downstream signalling (Fig. 3). MAX2 is an F‐box protein that forms part of a Skp‐Cullin‐F‐box (SCF^MAX2^) complex and targets the downstream repressors of karrikin and SLs signalling for degradation by ubiquitination (Soundappan *et al.*, 2015). These repressors include SUPPRESSOR OF MAX2 1 (SMAX1) and SMAX1‐LIKE (SMXL)2 that repress karrikin signalling (Stanga *et al.*, 2016), and SMXL6, SMXL7, and SMXL8 that repress SL signalling (Soundappan *et al.*, 2015). The other SMXLs (SMXL3, SMXL4 and SMXL5) have so far not been reported as repressors of karrikin or SL signalling (Wallner *et al.*, 2017). Research efforts are ongoing in order to gain more insight into the unique and common aspects of these two paralogous signalling pathways, karrikin and SL signalling (Hakoshima, 2018).

In addition to their effects on the germination of parasitic weeds *ex planta* (Nomura *et al.*, 2013), SL signalling facilitates the interaction of plants with arbuscular mycorrhizal fungi (AMFs) by triggering hyphal branching and the formation of the fungal hyphopodia, as has been shown in rice (*Oryza sativa*) using SL‐biosynthesis mutants (Kobae *et al.*, 2018). Another study that investigated the transcriptomic changes in germinated spores of the AMF *Gigaspora margarita* following SL (GR24) treatment suggests that SL induces expression changes in fungal genes involved in respiration, production of chitin oligosaccharides, and transcriptional reprograming in the fungus (Lanfranco *et al.*, 2018). SL‐specific effects on AMFs have also been reported; for example, dose–response analysis showed that sorgolactone and GR24 induced hyphal branching in *Gigaspora rosea* at 10^−13^ M, whereas GR7, which lacks the A‐ring, only stimulated branching at 10^−7^ M (Besserer *et al.*, 2006). Another study of SL structural specificity in AMF interactions demonstrated using the AMF *G. margarita* that intact AB‐ring structure is required for a high hyphal branching activity (Akiyama *et al.*, 2010). Furthermore, SLs are involved in the interaction of plant roots with nitrogen‐fixing bacteria (*Rhizobium*). There are reports of increased nodulation of alfalfa inoculated with *Sinorhizobium meliloti* following SL (GR24) treatment (Soto *et al.*, 2010; De Cuyper *et al.*, 2015). In pea also, SLs have been shown to enhance the development of infection threads during the interaction with *Rhizobium*, and endogenous SLs influence the number of nodules that are formed (Foo & Davies, 2011; McAdam *et al.*, 2017).

*In planta*, SL hormonal signals have been shown to inhibit axillary bud outgrowth (branching or tillering), in principle, independent of auxin signals (Brewer *et al.*, 2015), presumably by regulating the downstream expression of *BRANCHED 1* (*BRC1*). There are also reports of their involvement in moderating auxin canalization from buds to the main stem through the internalization of the auxin export protein (PIN1), thereby maintaining apical dominance (Hayward *et al.*, 2009; Shinohara *et al.*, 2013). Additionally, they play a role in the regulation of root architecture (Ruyter‐Spira *et al.*, 2011; Sun *et al.*, 2016). We hypothesize that the increasing scientific knowledge of the biological effects of SLs could translate into potential applications in agriculture (Box 2), as discussed in the following section.

**Fig. 4:**
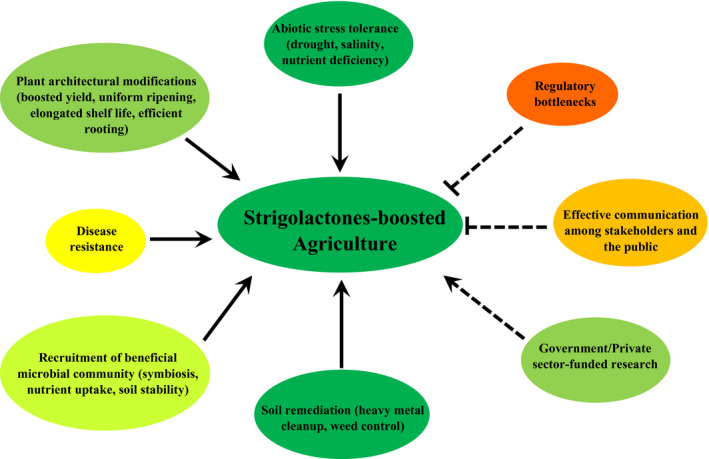
Illustration of potential application benefits (solid lines) of strigolactones in agriculture and the factors (broken lines) that are contributing to its potential realization, either positively (←) or negatively (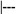). The colour hues of the panels represent the extent of knowledge and/or application on a field scale and the intensity of effects of the contributing factors (red, negative; green, positive).

Box 2 **Overview of requirements for strigolactone application (Fig. 4)**

There is currently little information on the field‐scale application of strigolactones (SLs), probably due to the high cost of SL synthesis and the knowledge gap on any potential off‐target environmental risks and/or side‐effects from SL degradation products. Targeted research is therefore needed to understand the specificity of various SLs, the importance of time of application, the possible dose‐dependent environmental risks above or belowground, SL effects on soil microbiota at the community level beyond the effects on a single species or genus, and so on. Importantly, SLs should be properly acknowledged and emphasized in studies in which the effects of SLs are evident, just like with other hormones. Also, collaboration among all stakeholders (government, private/industrial sector, and research institutes) is inevitable for the eventual large‐scale application of SL products. Finally, regulatory advocacy and public awareness may be necessary to provide law makers, farmers, and the general public with the correct information about SLs on which to base their judgements.

## Do strigolactones mediate the interaction with other soil‐dwelling organisms?

### Recruitment of beneficial microbial communities

In addition to the already established involvement of SLs in the interaction of plants with AMFs, recent studies have suggested that SLs can also affect the interaction with other soil (micro)organisms at both the individual and community levels (Lareen *et al.*, 2016; Schlemper *et al.*, 2017). For instance, in a nonfertile soil, the *Striga*‐resistant cv SRN‐39, which exudes a high orobanchol to 5‐deoxystrigol (5‐DS) ratio, recruits a different bacterial community than high 5‐DS/orobanchol‐exuding genotypes (Schlemper *et al.*, 2017). The dissimilarity in bacterial community recruitment observed by Schlemper *et al.* (2017) was mainly reflected in the abundance of *Comamonadaceae* and *Burkholderiaceae* families that were recruited significantly more by ‘SRN‐39’ than by other genotypes. This may suggest that these two bacterial families contribute to the *Striga* resistance of ‘SRN‐39’. The genetic basis for the unique SL profile of ‘SRN‐39’ and its resultant *Striga* resistance was further investigated using a recombinant inbred line population by Gobena *et al.* (2017). Interestingly, allelic deletion mutations in the *LOW GERMINATION STIMULANT 1* gene were shown to be responsible for the observed SL profile and resistance phenotype (Gobena *et al.*, 2017). Indeed, a potential ‘allelic’ control of SL biosynthesis and effects has also been demonstrated in tobacco (*Nicotiana tabacum*) using CRISPR/Cas9‐targeted mutation of the *CCD8* SL‐biosynthesis gene (Gao *et al.*, 2018). The two mutant alleles of the closely related *CCD8* genes (*NtCCD8A* and *NtCCD8B*) showed distinctive and differential gene‐expression levels in root tissues, and in response to exogenous auxin, respectively, suggesting that SL biosynthesis and function may be altered at the allelic level (Gao *et al.*, 2018). Also, in Arabidopsis, a mutant line of the SL‐biosynthesis enzyme CCD8 (*max4*) has been used to demonstrate SL effects on plant–microbial interactions at the community level (Carvalhais *et al.*, 2018). The authors showed that SLs influence the composition of fungal communities in the rhizosphere. The fungal taxa/family/species, *Epicoccum nigrum*, *Penicillium*, *Fibulochlamys chilensis*, *Herpotrichiellaceae*, *Mycosphaerella* and *Mycosphaerellaceae* were more abundantly recruited to the root rhizosphere of the wild‐type than the *max4* mutant, whereas some members of other families/taxa, including *Fusarium*, *Alternaria* and *Pleosporaceae*, were more abundant in the rhizosphere of *max4* (Carvalhais *et al.*, 2018), suggesting that SLs can possibly also repel harmful microbes like *Fusarium*. The notion that SLs may also attract pathogenic fungi like *E. nigrum* and *Mycosphaerella* should be treated with care, as the taxonomic assignment, without identification to the species level due to lack of sufficient sequence information, makes conclusions on the involvement and identity of such microbes only speculative. For instance, *Mycosphaerella* is a vast genus that includes various species that have not yet been ascertained as pathogenic (Crous *et al.*, 2009), making it unreliable to conclude that the recruited *Mycosphaerella* in Carvalhais *et al.* (2018) is pathogenic. On the other hand, *Epicoccum nigrum*, which has a taxonomic assignment to the species level, has been studied in depth to understand its intermicrobial associations, and there is a report of how it may be utilized as a biological control agent of other phytopathogens (de Lima Fávaro *et al.*, 2012). As a step towards understanding the mechanisms involved in these plant–microbial interactions, Belmondo *et al.* (2017) have used a fungal mutant screening approach to show that SL signal perception may induce the production of reactive oxygen species and other changes in the mitochondria of fungi. Also, the work of Lanfranco *et al.* (2018) highlighted earlier is a part of the efforts towards unravelling such mechanisms of interaction.

In view of our current understanding, we speculate that the response to SLs in microbes may vary per plant species depending on the SL profile produced. Indeed, several studies have shown that the biosynthetic pathway differences among plant species in the conversion of carlactonoic acid (CLA) influence the SL profile produced (Charnikhova *et al.*, 2017; Iseki *et al.*, 2018; Y. Zhang *et al.*, 2018). According to Iseki *et al.* (2018), sorghum converts CLA to 5‐DS and finally into sorgomol, whereas moonseed converts CLA directly to strigol without a 5‐DS intermediate. Similarly, in tomato (*Solanum lycopersicum*), CLA was reportedly converted directly to orobanchol through the action of an as‐yet‐unknown enzyme(s) (Y. Zhang *et al.*, 2018). Interestingly, a recent study has demonstrated that the previously unknown enzyme catalysing the direct conversion of CLA to orobanchol in cowpea and tomato is cytochrome P450 CYP722C (Wakabayashi *et al.*, 2019). In maize (*Zea mays*), oxidation, epoxidation, ring cleavage, and lactonization steps have been postulated to result in the unique SL zealactone from CLA (Charnikhova *et al.*, 2017).

As already described, several studies have tried to unravel the mechanisms underlying the relation between SL profile and microbial recruitment. Further research is needed to improve our understanding of these mechanisms and the SL‐based interaction of plants with the soil microbiome. This may aid the implementation of SLs in recruiting microbial communities of agronomic importance (Hartman *et al.*, 2018; Toju *et al.*, 2018; Bouwmeester *et al.*, 2019). Moreover, other potential indirect benefits of SLs in the soil, like phytoremediation, may be linked to its role in plant–microbe interactions (Wu *et al.*, 2011; Lenoir *et al.*, 2016), although more dedicated research is needed to evaluate the mechanisms underlying this effect and its feasibility for application.

### Defence against biotic agents

Though the foregoing emphasizes the potential benefits of SL‐based recruitment of (components of) the soil microbiome by plants, other beneficial agricultural implementations of SLs in the soil are the control of parasitic weeds and defence against diseases. Parasitic weeds pose a serious threat to agriculture, considering the long viability of their seeds in the soil and their spread to other agricultural fields (Rubiales *et al.*, 2018). In rice, for instance, annual economic losses resulting from parasitic weeds in Africa is estimated at USD 200 million, and this increases by USD 30 million per annum (Rodenburg *et al.*, 2016). As a potential measure to mitigate parasitic weeds, Screpanti *et al.* (2016) reviewed practical applications such as suicidal germination, which has been tested in tobacco to control *Orobanche ramosa* (Zwanenburg *et al.*, 2016) and in sorghum to control *S. hermonthica* (Samejima *et al.*, 2016). Suicidal germination is an SL‐based induction of the germination of parasitic weeds in the absence of a suitable host such that the weeds die and are eliminated from the soil before the cultivation of the crop. In terms of resistance to plant diseases, the role of SLs is far less understood. In tomato, SL biosynthetic mutants have been used to demonstrate the positive role of SL in plant defence against fungal pathogens (*Botrytis cinerea* and *Alternaria alternate*) and root‐knot nematode (*Meloidogyne incognita*), based on its cross‐talk with other hormones, like jasmonic acid, salicylic acid, and abscisic acid (ABA) (Torres‐Vera *et al.*, 2014; Xu *et al.*, 2019). On the other hand, results from studies on pea plant resistance to *Fusarium oxysporum* and *Pythium irregulare* show no involvement of SL in plant defence (Blake *et al.*, 2016; Foo *et al.*, 2016). In Arabidopsis, the beet cyst nematode, *Heterodera schachtii*, was used to demonstrate that the involvement of SLs in the attraction of plant‐cyst nematodes to the host may be MAX2‐dependent (Martinez *et al.*, 2019), suggesting a possible crosstalk with the karrikin paralogous pathway. These findings suggest that the role of SLs in defence may be specific for certain plant–pathogen combinations only, requiring further research to fully unravel the underlying mechanisms.

## How can strigolactones contribute towards plant response to environmental stress?

### Physiological response to stress

There is increasing evidence for a role of SLs in the response of plants to osmotic stresses, such as drought and salinity (Visentin *et al.*, 2016; Ma *et al.*, 2017). Using SL‐biosynthesis mutants (i.e. *max3* and *max4*), Ha *et al.* (2014) demonstrated in Arabidopsis that there is crosstalk between SLs and ABA in regulating abiotic stress responses. Physiologically, the SL mutant lines had a low germination rate, but also a poor stomatal regulation during stress that could be attributed to ABA insensitivity (Ha *et al.*, 2014). Similar ABA–SL crosstalk has been demonstrated in *Lotus japonica*, showing the ABA insensitivity of the SL‐biosynthesis mutant *Ljccd7* (Liu *et al.*, 2015). However, in contrast to the findings of Ha *et al.* (2014), another recent study in Arabidopsis did not show ABA insensitivity of stomatal closure in SL‐biosynthesis mutants relative to wild‐type: the SL‐biosynthesis mutants (*max3* and *max4*) in Kalliola *et al*. (2019) were as equally sensitive to ABA as the wild‐type. As an addition to this contrast, a drought experiment in rice using SL‐biosynthesis and signalling mutants also showed that both SL‐deficient (*d10* and *d17*) and insensitive (*d3*) mutants had a high ABA accumulation in the shoot, resulting in drought tolerance, whereas the mutant, *d27*, was deficient in ABA and susceptible to drought (Haider *et al.*, 2018). The overexpression of *OsD27* (which the authors speculate is perhaps in addition to SLs also involved in ABA biosynthesis) and its high expression in the SL‐deficient and insensitive mutants suggest that D27 may be involved in SL–ABA crosstalk. A further investigation of the aforementioned contrast using Arabidopsis single and double mutants of SL signalling (*max2*), ABA biosynthesis (*aba2*), and ABA guard cell signalling (*ost1*) in Kalliola *et al.* (2019) showed that a combined impairment of SL signalling and ABA biosynthesis or signalling led to higher stomatal conductance in the double mutants (i.e. an enhanced impairment of stomatal closure) than in the respective single mutants, pointing to a possible ABA‐independent/*MAX2*‐mediated stress response. This ABA‐independent (*MAX2*‐mediated) stress response, however, may be a concerted role of the strigolactones–karrikins paralogous pathways, since both molecules signal through MAX2. Also, microarray analysis of the *max2* mutant in Ha *et al.* (2014) revealed a downregulation of flavonoid biosynthesis‐related genes that are drought‐inducible in an ABA‐independent manner. Therefore, despite the unexpected contrasts between the findings from Ha *et al.* (2014) and those of Kalliola *et al.* (2019) and Haider *et al.* (2018), the results from all these studies suggest that SLs may contribute to abiotic stress responses both in concert with ABA and in parallel to it via the *MAX2*‐dependent signalling pathway.

Other reports re‐emphasize that the basis of the aforementioned ABA–SL crosstalk may be upstream of their respective biosynthesis pathways. Wang *et al.* (2018) demonstrated that the endogenous accumulation of ABA due to RNA interference silencing of *HvABA 8‐hydroxylase 1* and *3* in barley (*Hordeum vulgare*) resulted in a transcriptional downregulation of SL biosynthesis genes. Such ABA regulation of SL biosynthesis may depend on the direction of the reversible conversion of *all‐trans‐*β‐carotene to 9‐*cis*‐β‐carotene by *DWARF27*, since both hormones share *all‐trans‐*β‐carotene as a common precursor (Haider *et al.*, 2018; Wang *et al.*, 2018; Fig. 1). In tobacco also, the characteristics of the CRISPR/Cas9‐mutated alleles of the closely related *NtCCD8* biosynthetic genes (*NtCCD8A* and *NtCCD8B*) suggest that mutations in SL biosynthesis genes may contribute to ABA (in)sensitivity (Gao *et al.*, 2018). The loss‐of‐function tobacco mutants in Gao *et al.* (2018) differed in a point insertion (*NtCCD8A*) and a three‐point deletion (*NtCCD8B*), and the exogenous ABA treatment of these mutants resulted in a three‐fold boost in the gene expression of *NtCCD8B* but not *NtCCD8A*. Interestingly, however, the gene expression of *NtCCD8A* under phosphate starvation (nutrient stress) was six‐fold higher than that of *NtCCD8B*, suggesting that allelic variations in SL biosynthesis genes may also influence response mechanisms to different stresses.

In terms of the practicality for agricultural implementation of the aforementioned SL physiological roles on stomatal regulation, other studies have suggested that foliar spray is sufficient to induce SLs effects, thus circumventing the rigours of root treatment (Visentin *et al.*, 2016; Min *et al.*, 2019). In tomato, Visentin *et al.* (2016) grafted wild‐type (SC^WT^) and SL‐depleted (SC^SL^) scions on to wild‐type (RS_WT_) and SL‐depleted (RS_SL_) root stocks to investigate drought responses (Visentin *et al.*, 2016). Interestingly, drought stress in the wild‐type graft (SC^WT^/RS_WT_) induced the expression of SL biosynthetic genes in the shoot, which was similarly observed in the SL‐depleted root stock grafted on wild‐type scion (SC^WT^/RS_SL_) under irrigated conditions. The graft combinations with wild‐type scions were more drought tolerant than the graft with SLs‐depleted scion. This suggests that local SLs availability in the shoot may be critical for drought tolerance response. In fact, Visentin *et al.* (2016), using the mutant lines, showed that lack of SLs in the shoot limited the plants’ sensitivity to ABA‐induced stomatal closure, and exogenous SL application boosted this essential drought‐response phenotype. Also, foliar application of GR24 on grapevine seedlings subjected to polyethylene glycol treatment has recently been shown to alleviate drought stress through stomatal regulation (Min *et al.*, 2019). In agriculture, different genotypes of various crop species display varied levels of ABA sensitivity during drought – including delayed stomatal regulation in sensitive genotypes. Exogenous SL application may be used in the field to synchronize stomatal closure before water limitation results in a stress on the often‐sensitive crops. In essence, there is evidence of the application of SLs in maize fields leading to more effective drought tolerance compared with nontreated fields (Davidson, 2015). Nevertheless, further research is needed to understand the possibilities of combinatorial formulations of SLs and ABA that could be used in agriculture for synchronized stomatal regulations.

### Root development towards stress adaptation

Several reports have demonstrated the involvement of SLs in the regulation of plant root development, even though the specific effects may vary across species and conditions. For instance, lateral root development may be inhibited by SLs under optimal growing conditions, whereas during nutrient stress they are enhanced to facilitate nutrient uptake (Ruyter‐Spira *et al.*, 2011; Marzec & Melzer, 2018). A common observation among these reports, however, is the interaction of SLs with auxin, in which SLs play a superimposing regulatory role in modulating phenotypic response to the hormonal interplay (Ruyter‐Spira *et al.*, 2011; Kapulnik & Koltai, 2014; Sun *et al.*, 2019). In addition to its effects on lateral root development, SLs also positively regulate primary root length, seminal root length, root biomass, and root hair length and density (Ruyter‐Spira *et al.*, 2011; Kapulnik & Koltai, 2014). In a study using *PLEIOTROPIC DRUG RESISTANCE1* (*PDR1*)‐overexpressing lines in *Petunia hybrida*, it was shown that an optimization of SL transport could release the feedback inhibition on SL biosynthesis and boost plant rooting properties (Liu *et al.*, 2018). The overexpression of *PDR1*, an ABCG‐class transporter involved in SL transport to the shoot and exudation to the soil, resulted not only in the enhancement of root biomass and lateral root growth, but also induced root hair elongation (Liu *et al.*, 2018). In fact, the *P. hybrida PDR1*‐overexpressing lines showed a weaker auxin reporter intensity when compared with wild‐type, suggesting that the boosted endogenous SL levels may have influenced auxin distribution in the root, thus driving the concerted hormonal regulation of the root development in Liu *et al.* (2018). In terms of root hair formation and elongation, it is known that auxin transport from the root tip to the root hair zone is essential to trigger root hair formation, whereas a synergy of both auxin and ethylene pathways is essential in root hair elongation (Rahman *et al.*, 2002; Muday *et al.*, 2012; D. J. Zhang *et al.*, 2018). Interestingly, it has been reported that SLs regulate auxin‐efflux carriers like PIN1 that influence auxin levels in root cells and thus affect root hair growth, thereby introducing another layer of hormonal control of root hair development centred around SLs (Koltai *et al.*, 2010; Kapulnik *et al.*, 2011). Indeed, complex hormonal interactions between SL, auxin (IAA), cytokinin, ethylene, and karrikin pathways may all contribute to the eventual root architectural modifications that the plant can benefit from during environmental stress (Marzec *et al.*, 2013; Marzec & Melzer, 2018; Fig. 3). An additional role of SLs in these complex hormonal interactions that may be harnessed in nutrient‐poor soils and in organic agriculture to enhance nutrient uptake is the initiation of root symbiosis with nutrient‐fixing microbes during nutrient stress. The potential of this may be explored in seed‐propagated crops by seed treatment with SL formulations before planting in nutrient‐poor soils. The idea is to boost root development for specific soil environments such that crops thrive amidst a stressful environment. Hence, this approach may also be exploited in drought‐prone areas, so that crops develop increased root density early in the growing season before the onset of drought.

## How important is the effect of strigolactones on shoot architecture?

SL signalling via the receptor, D14, plays a significant role in the regulation of plant shoot architecture (Fig. 3). Among the degradation targets of SL signalling mediated by MAX2, SMXL7 was shown to be rapidly degraded by the synthetic SL GR24 (Soundappan *et al.*, 2015), and the features of SMXL6 were further reported to be similar to SMXL7 (Bennett *et al.*, 2016). A combined loss‐of‐function mutation of these SMXL genes (*SMXL6*, *7*, and *8*) suppressed shoot branching in *max2* (Soundappan *et al.*, 2015), indicating that the regulation of these SMXLs plays a pivotal role in shoot architectural modification. Furthermore, it has been shown with an *smxl6,7,8 max2* quadruple mutant that these SMXLs promote auxin transport and PIN1 accumulation in the stem, suggesting that SLs modulate shoot branching through their effect on auxin via these SMXLs (Soundappan *et al.*, 2015). The SMXLs also repress the expression of *BRC1* in axillary buds, thus releasing the suppression of axillary bud outgrowth by BRC1 (Soundappan *et al.*, 2015). BRC1 is known to act downstream of the SLs signalling pathway in some species like pea (PsBRC1) and Arabidopsis (AtBRC1) (Aguilar‐Martinez *et al.*, 2007; Braun *et al.*, 2012). A typical example of the agricultural implications of *BRC1* is the domestication of maize, in which the selection for less shoot branching in favour of yield is associated with the *TEOSINTE BRANCHED 1* (*TB1*) locus, a maize homologue of *BRC1* (Kellogg, 1997). The interaction between SLs and the *TB1* locus of maize in the regulation of shoot branching is, however, quite complex. Analysis of a maize SL‐biosynthesis (*Zmccd8*) and *tb1* mutant and the double mutant (*tb1‐Zmccd8*) revealed that although SLs may not completely regulate the *TB1* gene in maize, they have significant additive effects on shoot branching and plant height under limited *TB1* allele dosage (Guan *et al.*, 2012). The findings of Guan *et al.* (2012) also showed that, without SLs, maize ear length and diameter were significantly reduced and the tassel drooped. These architectural–phenotypic traits are essential for the productivity of the crop and may provide applications for SLs differing between species depending on the preferred trait. For instance, whereas branchless single stems are preferred in maize, the production of more tillers, as in rice and wheat (*Triticum aestivum*), may be of higher agronomic value, and tiller number may be associated with the production of less SLs (Wu *et al.*, 1998; Jamil *et al.*, 2012; Zhao *et al.*, 2019). With this understanding, an application of SL formulations on seedlings in the field may be used to influence shoot architecture to facilitate a preferential partitioning of metabolites to the yield organ. In the case of maize, for instance, there are reports of a 20% yield increase following SL foliar application in three field trials (Davidson, 2015). Also, in *Brassica napus*, there is evidence from a glasshouse study in growth chambers that GR24 application significantly increased plant biomass within 7 days of application (Ma *et al.*, 2017). Furthermore, there is emerging evidence of a potential role for exogenous SL application in late developmental stages of crop species. This was observed in grapevine both *in vitro* and in the field, whereby exogenously applied synthetic SLs interacted with exogenously co‐treated ABA (but not with endogenous ABA) to delay anthocyanin accumulation and ripening (Ferrero *et al.*, 2018). The latter finding suggests that there may be more levels and effects of SL hormonal interactions that can essentially be further investigated and harnessed to plan the timing of SLs application in the field. For instance, considering the food losses due to post‐harvest challenges in the handling of crop products that are delicate, field treatment with a combination of synthetic SL + ABA may delay ripening, which could be beneficial in enhancing the shelf life of farm products.

## Conclusions and perspectives

Variation in SLs (types, profiles, and concentrations) between plant species may differentially affect microbial communities. Research efforts to identify the respective SL biosynthesis pathways in distinct plant species would aid our understanding of such effects. It is essential to understand the roles that plant developmental stage, root architecture, environmental conditions, soil types, and so on play in the interactions between SLs and microbial communities. For this, the fundamental biology of SLs needs to be understood; for instance, the downstream signalling components in plants and perception in microbes, and the mechanisms of SL molecular and physiological interactions. The use of targeted mutation techniques (like CRISPR/Cas9) and population‐based studies could unlock the genetic basis of SL effects and potential for heritability. Key for this improved understanding is the use of pure SLs instead of racemic mixtures in SL studies, as different isomers may signal through different pathways (Scaffidi *et al.*, 2014). Furthermore, including functional assays, like protein‐level interactions in SL experiments, will ensure that each experiment is exhaustively utilized to improve our understanding of SL effects. For application purposes, the translation of the SL potential into agriculture will require that field trials are also conducted as a follow‐up to findings from controlled environments. This would require stereoselective synthesis of only biologically active configurations in order to reduce chemical pollution in the field.

In conclusion, many of the beneficial effects described herein are highly aligned with the sustainability criteria; for example: the better use of natural resources, such as soil nutrients; and the increase of crop resilience, particularly in relation to climate change threats. New SL‐based technologies will represent a substantial paradigm shift in the area of crop protection by moving from a classic pest/disease control approach to more of a crop enhancement effect that harnesses the soil potential.

## Author contributions

HJB, ADM and CS initiated the idea of the paper. EBA, TM and HJB reviewed the scientific literature. CS and ADM contributed on the industrial aspect of the manuscript.
